# TLR4 Gene Polymorphisms Interaction With *Ascaris* Infection in Severe RSV Bronchiolitis

**DOI:** 10.3389/fped.2022.876882

**Published:** 2022-04-27

**Authors:** Jefferson Antonio Buendía, Erika Fernanda Lindarte, Fernando P. Polack

**Affiliations:** ^1^Department of Pharmacology and Toxicology, School of Medicine, Research Group in Pharmacology and Toxicology (INFARTO), Universidad de Antioquia, Medellín, Colombia; ^2^Fundacion Infant, Buenos Aires, Argentina

**Keywords:** severe bronchiolitis, polymorphism, respiratory syncytial virus, *Ascaris*. suum, Colombia

## Abstract

**Introduction:**

The identification of gene-environment interactions allows the recognition of groups with higher risk of morbidity. This study evaluated the interaction between the presence of TLR4 gene polymorphisms and *Ascaris* infection with severe bronchiolitis in a tropical Colombian region.

**Methods:**

We included all infants younger than 24 months hospitalized due to bronchiolitis in Hospital centers in the county of Rionegro, Colombia. To identify interaction between severe bronchiolitis and presence of TLR4 polymorphisms and *Ascaris* infection, we used log-binomial regression.

**Results:**

Four hundred and seventeen infants were hospitalized due to bronchiolitis, of which 115 (27%) had severe bronchiolitis. In infants with respiratory syncytial virus (RSV) acute infection and positive anti-Ascaris IgE, TLR4 Asp299Gly was associated to low risk of severe bronchiolitis (OR 0.09, CI 95% 0.01–0.48). Conversely, in infants RSV negative with negative anti-Ascaris IgE, TLR4 Asp299Gly was associated with an increased risk of severe bronchiolitis (OR 14.5, CI 95% 2.2–96).

**Conclusion:**

In our population there is an interaction between the presence of severe bronchiolitis, TLR4 Asp299Gly and Ile399Thr polymorphisms, anti-Ascaris IgE levels and RSV. This association should be evaluated in other populations to elucidate its role in the pathogenesis of severe bronchiolitis.

## Introduction

Respiratory syncytial virus (RSV) bronchiolitis is the most important cause of lower respiratory tract infection in children worldwide (1, 2). The disease places a substantial clinical and economic burden, not only on healthcare systems, but also on families and society, mainly in low-to- middle-income countries (LMICs) where more than 90% of the deaths occur (3–6). The treatment consists only in respiratory and hemodynamical support since there is no a specific intervention or vaccination yet (7). Identifying patients at higher risk of morbidity and mortality is essential to plan preemptive strategies in the future. Different determinants of bronchiolitis severity have been described, among them RSV infection, smoking, pre-existing diseases, absence of breastfeeding (8).

A gene-environment interaction as documented between toll like receptor 4 (TLR4) D299G single nucleotide polymorphism and environmental endotoxin exposure reveals a possible T helper 2 (Th2) polarizations in patients with severe RSV bronchiolitis, which may become useful for early identification of these patients at risk of severe RSV disease (9). However, in tropical countries, in addition to exposure to endotoxin, the presence of *Ascaris lumbricoides* infection has been associated with a Th2 profile in asthmatic or recurrent wheezing children (10). Although, both environmental stimuli may share similar immunological pathways, the gene-environment interaction between TLR4 polymorphisms and infection with *A. lumbricoides* has not been evaluated. Previously, we made a first report analyzing clinical and sociodemographic risk factors associated with severe bronchiolitis in a retrospective cohort of children with bronchiolitis <2 years of age in Rionegro, Colombia (8). In this study and, using the same population, we study aimed to evaluate the association between the presence of RSV bronchiolitis with TRL4 polymorphisms and *Ascaris* infection in infants under 2 years old.

## Methods

The methodology and characteristics of this population were reported previously (8). We included all children with bronchiolitis <2 years old in two hospital centers in the municipality of Rionegro Colombia, between January 2019 to December 2019. All infants diagnosed with bronchiolitis, according to the national clinical guideline of bronchiolitis, were included (8). We excluded infants who did not present lower respiratory infection or positive bacterial cultures on admission or confirmed whooping cough. The Institutional Review Board of the University of Antioquia approved the study protocol (No. 18/2015).

We collected sociodemographic, medical, and clinical variables from medical records in the hospital centers. All variables that were not fully documented in the clinical history were obtained by directly interviewing the parents or caregivers in person or by telephone, previous informed consent to the responsible for the children. Severe bronchiolitis was identified in patients who had an increased respiratory rate, retractions, and oxygen saturation of 90% or less (11). Nasopharyngeal aspirate (NPA) was taken during the first 48 h in the emergency unit using a standardized technique. RSV was confirmed using direct immunofluorescence. There was no data available for other viruses. ImmunoCAP (Thermo Fisher Scientific) was used to measure the levels of specific IgE antibodies against *Ascaris* spp. where it was considered positive when the specific IgE levels were equal to or greater than 0.35 kUA/L. DNA was obtained from whole blood samples using the Gentra Puregene kit (Gentra Systems) and DNA was quantified using a Beckman Coulter DU640 spectrophotometer. The REPLI-g kit (QIAGEN) was used for the amplification of the DNA samples. The TLR4 Asp299Gly and Ile399Thr polymorphisms were evaluated by allelic discrimination using Applied Biosystems assays that had probes with specific fluorophores for each of the polymorphisms. The thermal profile for PCR consisted of an initial denaturation at 95°C for 10 min, followed by 35 cycles at 92°C for 15 s and 60°C for 1 min. An ABI 7000 (Applied Biosystems) was used for PCR amplification and subsequent characterization of the genotypes for each polymorphism. Controls were used for the correct identification of genotypes in patient samples.

### Statistical Analysis

We analyzed the differences of continuous variables using the unpaired *t*-test or Wilcoxon's signed-rank test, whichever was appropriate, categorical variables were analyzed using the chi-square test or Fisher's exact test. We included only in the final model variables associated with severe bronchiolitis with values of *P* < 0.2 or that changed the effect estimate by over 10% after their inclusion, to identify factors independently associated with severe bronchiolitis. We also included in the model variables widely known and related to severe bronchiolitis such as age, smoking at home and exclusive breastfeeding (8, 12). A log-binomial regression model was performed with a backward elimination method, used with a *P*-value of 0.05 as the limit value for the model entry (7). Hosmer–Lemeshow test was used to evaluate he goodness of fit of the model. Two-tailed, and the significance level of *P* < 0.05 were used in all statistical tests were. All data were analyzed with Statistical Package Stata 15.0 (Stata Corporation, College Station, TX).

## Results

### Population Characteristics

During the study period, 417 infants with bronchiolitis were included. Table 1 shows the clinical characteristics of the infant population evaluated. Sixty-six percentage of the patients were <6 months, most of them males (60%), RSV was detected in 200 patients. TLR4 Asp299Gly (AG or AA) was present in 59 patients (14%), while TLR4 Ile399Thr (CC or CT) was present in 286 patients (68%). All the alleles of the TLR4 Asp299Gly and Ile399Thr polymorphisms were in Hardy-Weinberg equilibrium. Positive anti-Ascaris IgE levels (>0.35 kU/L) were observed in 74 patients (18%).

**Table 1:** Univariate analysis of demographic features and clinical information associated with severe bronchiolitis.

**Variable. *N* (%)**	***n* (%)**	**OR (CI 95%)**
Age <6 month	277 (66)	1.00 (0.99–1.00)
Male, *n* (%)	251 (60)	1.05 (0.67–1.62)
Premature birth	81 (19)	0.67 (0.40–1.13)
Comorbidities (CHD or neurological)	20 (4.81)	1.57 (0.51–4.82)
BPD	17 (4.09)	0.41 (0.15–1.10)
Atopy	17 (4.09)	0.92 (0.31–2.68)
Previously hospitalization by bronchiolitis	30 (7.21)	1.59 (0.63–4.00)
Exposure to cigarette smoking	49 (12)	2.53 (1.10–5.81)
Complete vaccination for age	415 (99)	0.67 (0.42–1.11)
Exclusive maternal breastfeeding for at least 6 month	102 (24)	0.96 (0.58–1.58)
O_2_ supportive, *n* (%)	347 (83)	16.5 (8.77–31.2)
Leucocytosis (>15,000/mm^3^)	51 (12)	1.02 (0.53–1.97)
RSV positive	200 (48)	1.77 (1.14–2.74)
TLR4 rs4986790 (AG/GG)	59 (13)	0.84 (0.47–1.51)
TLR4 rs4986791 (CT/TT)	286 (68)	1.10 (0.71–1.73)
Positive anti-Ascaris IgE levels (>0.35 kU/L)	74 (18)	2.79 (1.58–4.92)
Increased C-reactive protein (>4 mg/L)	327 (78)	1.76 (0.97–3.20)
Length of hospital stay, median (range)	3.68 (0.74–29)	1.02 (0.98 1.07)
PICU	55 (14)	0.93 (0.49–1.74)

*CHD, congenital heart disease; BPD, bronchopulmonary dysplasia; PICU, pediatric intensive care unit admission; RSV, respiratory syncytial virus*.

#### Univariable Analysis

Among all 417 infants, 115 (27%) had severe bronchiolitis. Exposure to smoking in home (OR 2.53, CI 95%, 1.10–5.81), positive anti-Ascaris IgE levels (OR 2.79, CI 95% 1.58–4.92), RSV (OR 1.77, CI 95% 1.14–2.74), crepitation 3.06 (OR, CI 95% 1.19–5.23), and pneumonia (OR 2.54, CI 95% 1.31–4.89) were associated with severe bronchiolitis, Table 1.

#### Multivariable Analysis

After modeling, we detected a statistically significant interaction between severe bronchiolitis and TRL4-IgE Ascaris in RSV-positive (*P* = 0.000) and non-RSV-positive children (*P* = 0.000). In infants with positive anti-Ascaris IgE levels, TLR4 Asp299Gly was associated with low risk of severe bronchiolitis independent of the presence or absence of RSV infection. Conversely, in infants with negative anti-Ascaris IgE levels, TLR4 Asp299Gly was associated with higher risk of severe bronchiolitis independent of the presence or absence of RSV infection (see Table 2).

**Table 2:** OR for severe bronchiolitis: association with TLR4 mutation according to anti-Ascaris IgE Levels and RSV.

**Environment**	**SNP Asp299Gly**	***n*/*N* (%)**	**Crude**	Adjusted*	***P*-Value**	*P*-Value**
			**OR (CI 95%)**			
**RSV positive**						
Positive anti-Ascaris IgE levels	Yes	2/128 (1.5%)	0.09 (0.01–0.50)	0.09 (0.01–0.48)	0.005	
	No	126/128 (98.5%)	1.00	1.00		<0.001
Negative anti-Ascaris IgE levels	Yes	16 /26 (61.5%)	3.22 (0.25–40)	2.88 (0.14–57)	0.488	
	No	10/26 (38.5%)	1.00	1.00		
**RSV negative**						
Positive anti-Ascaris IgE levels	Yes	2/102 (2%)	0.05 (0.07–0.46)	0.04 (0.05–0.48)	0.005	
	No	100/102 (98%)	1.00	1.00		<0.001
Negative anti-Ascaris IgE levels	Yes	24 /33 (72.7%)	13 (2.43–73)	14.5 (2.20–96)	0.005	
	No	9/33 (27.3%)	1.00	1.00		

*Logistic regression model, adjusted for age, smoking at home, exclusive maternal breastfeeding for at least 6 month.

**For interaction between TLR4 mutation and anti-Ascaris IgE levels.

## Discussion

Our study identifies an interaction between severe bronchiolitis, TRL4, anti-Ascaris IgE levels and RSV. This interaction has been previously reported with bacterial endotoxin exposure (14). Caballero et al. (9), reported a significant association between TLR4 Asp299Gly and the environment with different levels of endotoxins even after adjusting for risk factors that have an effect on the severity of RSV bronchiolitis. In this study patients with high endotoxin levels, TLR4 Asp299Gly mutation was associated with less risk of severe bronchiolitis (OR 0.20 CI 95% 0.06–0.64), while in infants with low endotoxin levels there was a tendency of higher risk of severe bronchiolitis (OR 8.96 CI 95% 0.98–81.6). In our study we observed a similar result with anti-Ascaris IgE levels. Previously evidence has demonstrated that *A. lumbricoides* induces an enhanced Th2-biased immune response that cause symptoms in susceptible individuals mediated by cross-reactivity among components of both sources intensifies the Th2 response associated to allergens such as tropomyosins (13).

*Ascaris lumbricoides* induce the adaptive immune response through its interaction with the extracellular domain of the TLR4 receptor(cite). Our study shows that this effect is regulated by the presence of variants in the TLR4 gene, such as TLR4 Asp299Gly which have been associated with a loss of function of this receptor in the plasma membrane according to previous studies (15). Caballero revealed that children with severe RSV exhibited a high GATA3/T-bet levels, which manifested as a high IL-4/IFN-γ in respiratory secretions. The IL-4/IFN-γ present in children with severe RSV is indicative of Th2 polarization. Murine models of RSV infection showed that endotoxin exposure, the Tlr4 genotype, and Th2 cell polarization influence disease phenotypes (9). Due to the similarity of the findings, it is possible that the mechanisms by which the bacterial endotoxin generates this antagonistic interaction are the same as those occurring in Ascaris infection, a hypothesis to be studied further.

Our study has some limitations. We were unable to include other variables such as environmental pollution and other genetic factors considered important for susceptibility to the disease, and residual confounding cannot be excluded. Second, the study was conducted in a reference hospital center, so the patients included represent the high spectrum of severity. Despite the above, the similarity of our population regarding clinical characteristics, risk factors and seasonality of bronchiolitis in our country coincides with the characteristics of other populations also evaluated for this disease, which suggests strength and consistency in our results (16). Given the retrospective nature would be possible bias due to missing data. However, in all patients the data was collected in electronic records or directly to the patient in outpatient consultations that these patients have in the hospital after their hospitalization. We concluded that in our population there is an interaction between the presence of severe bronchiolitis, TLR4 Asp299Gly and Ile399Thr polymorphisms, anti-Ascaris IgE levels and RSV. This association should be evaluated in other populations to elucidate its role in the pathogenesis of severe bronchiolitis.

## Data Availability Statement

The original contributions presented in the study are included in the article/supplementary material, further inquiries can be directed to the corresponding author.

## Ethics Statement

The studies involving human participants were reviewed and approved by Institutional Review Board of the University of Antioquia approved the study protocol (No. 18/2015). Written informed consent to participate in this study was provided by the participants' legal guardian/next of kin.

## Author Contributions

All authors listed have made a substantial, direct, and intellectual contribution to the work and approved it for publication.

## Conflict of Interest

The authors declare that the research was conducted in the absence of any commercial or financial relationships that could be construed as a potential conflict of interest.

## Publisher's Note

All claims expressed in this article are solely those of the authors and do not necessarily represent those of their affiliated organizations, or those of the publisher, the editors and the reviewers. Any product that may be evaluated in this article, or claim that may be made by its manufacturer, is not guaranteed or endorsed by the publisher.

## Abbreviations

CHD: congenital heart disease
BPD: bronchopulmonary dysplasia
PICU: pediatric intensive care unit admission
RSV: respiratory syncytial virus
NPA: nasopharyngeal aspirate.
